# Development of cardiovascular and all-cause mortality risk prediction models for maintenance hemodialysis patients based on metabolomics

**DOI:** 10.1186/s12882-025-04291-0

**Published:** 2025-07-10

**Authors:** Lian-Lian You, Cui Dong, Zhi-Hong Wang, Shuang Zhang, Yu Zhang, Ting-Ting Kuai, Jia Xiao, Shu-Xin Liu, Qing-Cheng Zeng

**Affiliations:** 1 School of Maritime Economics and Management, Dalian Maritime University, No.1, Linghai Road, Dalian, Liaoning 116026 P. R. China; 2 Department of Nephrology, Central Hospital of Dalian University of Technology, No.826, Xinan Road, Dalian, Liaoning 116033 P. R. China; 3 Dalian Key Laboratory of Intelligent Blood Purification, Central Hospital of Dalian University of Technology, Dalian, China

**Keywords:** All-cause mortality, Cardiovascular mortality, Maintenance hemodialysis, Metabolomics, Prediction model

## Abstract

**Introduction:**

The outcome of maintenance hemodialysis (MHD) remains poor, with cardiovascular death accounting for more than half of all-cause death cases. In this study, cardiovascular mortality and all-cause mortality prediction models were developed to investigate the predictive role of metabolites in MHD patients.

**Methods:**

Clinical and metabolomics data of 135 hemodialysis patients from a single center were collected with a 6-year follow-up. Univariate Cox regression and random forest were respectively applied to preliminarily screen clinical and metabolomics characteristics, followed by multivariate Cox regression for identifying features predicting cardiovascular or all-cause mortality. Multivariate Cox proportional regression risk models were constructed using clinical, metabolomics, and combined features. Subgroup survival differences were compared via risk score stratification.

**Results:**

The combined model showed significant superiority in predicting cardiovascular mortality (3-year AUC = 0.901, 5-year AUC = 0.876), surpassing the clinical-only model (0.868/0.826) and metabolomics-only model (0.659/0.641). For all-cause mortality, the combined model demonstrated modest improvement (0.859/0.834) but still outperformed the metabolomics model (0.534/0.653). Thirty 5-fold cross-validations confirmed stable performance. High-risk groups had significantly higher cumulative mortality than low-risk groups (*p* < 0.0001).

**Conclusion:**

The metabolomics-alone model showed limited predictive performance, but its synergistic integration with clinical indicators further improved the predictive performance of mortality risk models, particularly for cardiovascular mortality.

**Supplementary Information:**

The online version contains supplementary material available at 10.1186/s12882-025-04291-0.

## Introduction

Maintenance hemodialysis (MHD) is the most widely used treatment method for patients with end-stage renal disease (ESRD) in China, accounting for 86% of dialysis patients in 2015 [1]. Despite the continuing progress in hemodialysis therapy, the mortality remains unacceptably high among MHD patients. As reported by the United States Renal Data System, the adjusted mortality rate in patients receiving hemodialysis was 159.3 per thousand person-years in 2019, and over half of known deaths were related to cardiovascular disease (CVD) causes [2]. However, there is no molecular biomarker with high sensitivity and specificity that can be employed for the early diagnosis of cardiovascular outcomes in MHD patients.

As an important branch of systems biology, metabolomics is widely used in the fields of disease mechanisms, gene function, diagnosis, and prognosis prediction, among others, making precision medicine possible [3]. It has been shown that CVDs are accompanied by disorders of the cardiac metabolism, which affect fatty acid, glucose, amino acid, and ketone body metabolism processes [4, 5]. A metabolomics study based on targeted quantitative liquid chromatography/mass spectrometry demonstrated a positive association between trimethylamine N-oxide concentrations and cardiovascular events in hemodialysis patients [6]. In addition, Hu et al. found that the odds of cardiovascular death were higher with higher levels of several lipid metabolites, an amino acid metabolite (2-hydroxybutyrate/2-hydroxyisobutyrate), and phosphate [7]. These studies suggest the potential of metabolomics for predicting CVD in MHD patients.

The risk prediction model is a mathematical tool used to predict the probability of an endpoint event. Most of the previous prediction models for cardiovascular events or deaths in patients with ESRD developed through survival analysis, regression models, and machine learning are based on clinical indicators, such as medical record information, laboratory tests, examinations, etc [8–10]. However, these clinical risk factors cannot fully account for the high risk of death in dialysis patients [11]. The present study is the first to propose combining clinical indicators with metabolomic markers to construct models for predicting cardiovascular and all-cause mortality, and these models were presented as nomograms with the aim of exploring the role of metabolomics in predicting the risk of cardiovascular and all-cause mortality in MHD patients.

## Materials and methods

### Study design and patient selection

The present investigation was a single-center retrospective cohort study of patients on MHD. Cardiovascular death and all-cause death served as the key outcomes. Baseline demographics, comorbid medical, clinical laboratory, and metabolomics characteristics for the cohort were extracted and analyzed.

**Fig. 1:**
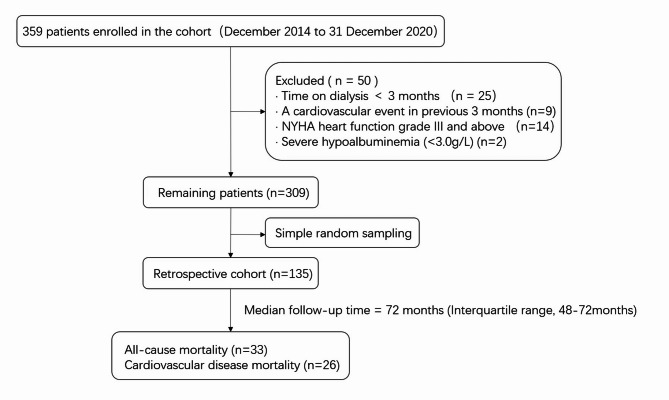
Flowchart of patient inclusion and study design for MHD patients

Patients were recruited at the Blood Purification Center of the Central Hospital of Dalian University of Technology (Dalian, China) in December 2014. The follow-up period was six years, lasting until December 31, 2020. The inclusion criteria were patients aged over 18 years, in stable condition, using a standard bicarbonate dialysate for dialysis, receiving treatment three times a week (4 h per session), and using arteriovenous fistulas. Patients were excluded if they had cardiovascular events within three months prior to enrollment (including acute myocardial infarction, unstable angina, acute heart failure, malignant arrhythmia, cerebrovascular disease, and thrombosis or embolism of the peripheral arteries), New York Heart Association heart function grade III and above, severe hypoalbuminemia (< 30 g/L), dialysis vintage of ≤ 3 months. Ultimately, 135 participants were included in the study (Fig. 1). The study was approved by the Ethics Committee of the Central Hospital of Dalian University of Technology, and waiver of informed consent was obtained.

### Outcome assessment

Cardiovascular and all-cause death were respectively the primary analysis outcomes during the follow-up period. Mortality outcomes were determined based on the information recorded using the therapy support suite (version 2.0, Treatment Support Kit, Baden-Humboldt, Germany) and B-soft Enterprise Application Portal (version 5.5, B-soft CO., LTD, Hangzhou, China) software. Cardiovascular deaths included sudden cardiac death, coronary events, heart failure, arrhythmias, and cardiomyopathy. For each participant, the time to event was calculated from the date of entry into the study to the date of the first study event (mortality), the date of withdrawal from the study, the date of kidney transplantation, or the date of study completion, whichever came first.

### Measurement of covariates

Age, gender, height, weight, blood pressure, dialysis vintage, dialysis modality, primary disease, complications, and medication use data were obtained from hospital records in the BS-EAP (version 5.5, B-soft CO., LTD) software. Venous blood samples were drawn from hemodialysis patients before and after the midweek dialysis session. One portion of the plasma sample at the beginning of the dialysis was stored at −80 °C until sample preparation for the plasma metabolite assay. The other portion was immediately shipped out for routine laboratory analysis. Laboratory data included hemoglobin (Hb), platelet (PLT), alanine transaminase (ALT), albumin (ALB), alkaline phosphatase (ALP), creatinine (Cr), potassium (K), sodium (Na), calcium (Ca), phosphorus (P), parathyroid hormone (PTH), and blood urine nitrogen (BUN) levels before and after dialysis. To avoid dilution when obtaining post-dialysis urea, the ultrafiltration rate was set to zero and the blood pump rate was reduced to 100 mL/min. The sample was then drawn from the arterial needle tubing 15 s after reducing the blood flow [12]. Dialysis adequacy was determined using the single-pool Kt/Vurea (spKt/v), which was calculated based on the second generation equation of Daugirdas utilizing pre- and post-hemodialysis blood urea levels as follows: spKt/v = − ln(*R* − 0.008 × t) + [(4 − 3.5 × R) × UF/W], where R is the ratio of post-dialysis to pre-dialysis serum urea nitrogen concentration, t is the dialysis session length in h, UF is the ultrafiltrate volume in L, and W is the post-dialysis weight in kg [13].

### Measurement of plasma metabolites

After sample preparation, non-targeted metabolomics technology based on capillary electrophoresis (CE; G7100A, Agilent, USA) combined with time-of-flight mass spectrometry (TOF/MS; G6224A, Agilent, USA) was used to analyze plasma metabolites. The CE system was controlled by the Chem Station software (B.04.03, Agilent), and the TOF/MS system was managed using the Mass Hunter Workstation software (B.04.00, Agilent). A fused-silica capillary (80 cm × i.d. 50 μm; Human Metabolome Technologies, Inc., Japan) was used to separate the metabolites. The capillary temperature was maintained at 20 °C. Methanol/water solution (1:1, V/V) with 0.1 µM hexakis (2,2-difuoroethoxy) phosphazene was used as a sheath liquid. Data acquisition was performed under electrospray ionization in a full scan mode. The capillary voltage was set to 27 kV in the positive mode and a 30-min separation was used for each sample. The reconstituted sample was then injected for 3 s at 50 mbar (i.e., 3 nL). The capillary voltage was set to 30 kV in the negative mode and a 40-min separation was used for each sample. The reconstituted sample was injected for 25 s at 50 mbar (i.e., 25 nL). Mass parameters were as follows: nebulizer pressure was set to 5 psig, nitrogen flow was set to 7 L/min, dry gas temperature was set to 300 °C, Oct RFV was set to 650 V, skimmer was set to 50 V, capillary voltage was set to 4 kV (+) and 3.5 kV (−), the fragmentor was set to 105 V (+) and 125 V (−), and mass ranges were 60–1000 (+) and 50–1000 (−).

### Statistical analyses

The baseline characteristics of participants across outcome groups were presented as follows: normally distributed continuous variables as mean ± standard deviation, non-normally distributed continuous variables as median (interquartile range, IQR), and categorical variables as frequencies (percentages). Statistical analyses were performed using t-test for normally distributed continuous variables, Mann-Whitney U test for non-parametric continuous variables, and Chi-square test for categorical variables. Data with missingness > 10% were excluded. Median imputation was applied to non-normally distributed continuous variables with missing values, whereas metabolite missing data were handled through random forest imputation. A two-sided p-value < 0.05 was considered statistically significant.

The feature screening was conducted in a hierarchical manner. First, univariable Cox regression was conducted for each clinical variable individually, and variables with a statistically significant association (*p* < 0.05) were retained. Subsequently, a random survival forest model was applied to rank the importance of the metabolites, and the top 10 metabolites based on importance rankings were selected. Finally, the screened clinical variables and metabolites were jointly incorporated into a multivariable Cox proportional hazards regression model to calculate hazard ratios (HRs), 95% confidence intervals (CIs), and corresponding p-values, establishing predictive models for CVD and all-cause mortality.

To evaluate the predictive efficacy of metabolomic biomarkers, three Cox proportional hazards regression models were systematically developed: a clinical characteristic-based model (clinical model), a metabolic signature-driven model (metabolomics model), and a composite model integrating both modalities (combined model). To rigorously control the overfitting risk and ensure the robustness of validation outcomes, 30 rounds of five-fold cross-validation are performed for internal validation. The area under the curve (AUC) serves as a metric to quantitatively evaluate the discriminative capacity of these models. For each developed model, the median value of the generated risk scores is used as the cutoff point to stratify the cohort into high-risk and low-risk subgroups. The cumulative incidence function is then applied to compare the survival differences between the two subgroups.

All statistical analyses were performed using R software (version 4.1.2, R Foundation for Statistical Computing, Austria).

## Results

### Baseline characteristics of participants

This study included 135 patients with a mean age of 49.41 ± 12.42 years and a median dialysis vintage of 74.0 (IQR: 53.5–107.0) months. During the median follow-up period of 72.0 (IQR: 48.0–72.0) months, 33 all-cause deaths (24.4%) were recorded, including 26 deaths (19.3%) from CVD. Baseline clinical characteristics of the cohort are listed in Table 1. A comparison between the cardiovascular death group and the survival group revealed significant differences (*p* < 0.05) in eight clinical indicators: age, Hb, PLT, ALP, PTH, systolic blood pressure (SBP), high-flux hemodialysis (HFD) modality, and CVD. Notably, when comparing the all-cause death group with survivors, diabetes mellitus (DM) emerged as an additional significant factor (*p* < 0.05), along with the aforementioned eight variables.

**Table 1:** Baseline clinical characteristics of the study population

Variables	All (*n* = 135)	Survival (*n* = 102)	CVD death (*n* = 26)	All-cause death (*n* = 33)	*P* survival vs. CVD death	*P* survival vs. All-cause death
Gender (Male)	88 (65.19%)	62 (60.78%)	20 (76.92%)	26 (78.79%)	0.126	0.059
Age (years)	49.41 ± 12.42	46.78 ± 11.58	58.19 ± 12.78	57.55 ± 11.53	< 0.001^*^	< 0.001^*^
Dialysis vintage (months)	74.00 (53.50–107.00)	73.50 (53.00–94.00)	90.00 (59.50–126.00)	81.00 (56.00-126.00)	0.057	0.206
BMI (kg/m^2^)	22.53 (20.32–25.92)	22.78 (20.21–26.24)	21.25 (20.55–24.49)	21.85 (20.45–25.48)	0.448	0.699
Hb(g/L)	113.00 (105.00-118.50)	114.00 (108.00-119.00)	107.00 (100.00-113.00)	105.00 (100.00-113.00)	0.021^*^	0.014^*^
PLT (*10^9^/L)	186.00 (154.50-217.50)	195.00 (158.00-225.75)	178.50 (121.75-198.75)	174.00 (126.00-193.00)	0.048^*^	0.009^*^
ALT (U/L)	12.00 (8.00–16.00)	12.00 (8.00–17.00)	12.00 (7.25-15.00)	12.00 (8.00–16.00)	0.597	0.872
ALB (g/L)	41.39 ± 2.22	41.59 ± 2.22	40.95 ± 2.28	40.79 ± 2.16	0.191	0.071
ALP (U/L)	89.00 (70.50–117.00)	88.00 (67.50–111.00)	111.00 (79.75–194.00)	109.00 (77.00-173.00)	0.007^*^	0.008^*^
BUN (mmol/L)	27.01 (23.84–29.58)	26.43 (23.80-29.32)	28.02 (24.90-30.66)	28.21 (24.30–29.70)	0.183	0.200
Cr (µmol/L)	1033.00 (908.00-1225.00)	1072.00 (923.25-1245.75)	966.50 (832.00-1111.00)	967.00 (834.00-1115.00)	0.107	0.086
K (mmol/L)	5.04 (4.46–5.72)	5.03 (4.46–5.58)	5.03 (4.67–5.80)	5.06 (4.67–5.89)	0.702	0.472
Na (mmol/L)	134.39 ± 3.22	134.45 ± 3.20	134.44 ± 2.91	134.21 ± 3.32	0.993	0.709
Ca (mmol/L)	2.39 (2.25–2.49)	2.38 (2.25–2.49)	2.37 (2.25–2.46)	2.39 (2.27–2.47)	0.745	0.986
P (mmol/L)	2.08 (1.73–2.45)	1.99 (1.72–2.38)	2.17 (1.90–2.60)	2.20 (1.87–2.60)	0.171	0.168
spKt/v	1.34 (1.21–1.51)	1.38 (1.21–1.53)	1.31 (1.24–1.41)	1.28 (1.20–1.40)	0.687	0.312
PTH (pg/mL)	351.90 (183.40-759.50)	322.80 (149.72-652.85)	668.55 (337.12-1160.50)	614.30 (351.90-1162.50)	0.007^*^	0.004^*^
SBP (mmHg)	150.00 (139.50–160.00)	150.00 (130.75–160.00)	160.00 (141.75-169.25)	160.00 (150.00-170.00)	0.028^*^	0.011^*^
DBP (mmHg)	84.00 (80.00–91.00)	81.50 (80.00–90.00)	90.00 (80.00-100.00)	90.00 (80.00-100.00)	0.078	0.100
DN (%)	9 (6.67%)	4 (3.92%)	3 (11.54%)	5 (15.15%)	0.298	0.065
DM (%)	15 (11.11%)	7 (6.86%)	5 (19.23%)	8 (24.24%)	0.120	0.015^*^
Hypertension (%)	116 (85.93%)	84 (82.35%)	25 (96.15%)	32 (96.97%)	0.145	0.070
CVD (%)	27 (20.00%)	11 (10.78%)	11 (42.31%)	16 (48.48%)	< 0.001^*^	< 0.001^*^
Renal transplantation (%)	6 (4.44%)	4 (3.92%)	2 (7.69%)	2 (6.06%)	0.770	0.974
Statins use (%)	11 (8.15%)	6 (5.88%)	5 (19.23%)	5 (15.15%)	0.076	0.185
Antihypertensive drugs use (%)	95 (70.37%)	71 (69.61%)	20 (76.92%)	24 (72.73%)	0.463	0.733
HFD (%)	42 (31.11%)	37 (36.27%)	3 (11.54%)	5 (15.15%)	0.015^*^	0.023^*^
IDH (%)	6 (4.44%)	5 (4.90%)	0 (0.00%)	1 (3.03%)	0.559	1.000
3-year mortality (%)	17 (12.59%)	0 (0.00%)	15 (57.69%)	17 (51.52%)	NA	NA
5-year mortality (%)	30 (22.22%)	0 (0.00%)	24 (92.31%)	30 (90.91%)	NA	NA

ALB: albumin; ALP: alkaline phosphatase; ALT: alanine aminotransferase; BMI: body mass index; BUN: blood urine nitrogen; CVD: cardiovascular disease; DBP: diastolic blood pressure; DM: diabetes mellitus; DN: diabetic nephropathy; Hb: Hemoglobin; HFD: high-flux hemodialysis; IDH: intradialysis hypotension; NA: not applicable; PLT: platelet; PHT: parathyroid hormone; SBP: systolic blood pressure; spKt/v: single-pool Kt/V

### Features selection

Multivariable Cox regression analysis incorporating clinically significant variables (*p* < 0.05) from univariable Cox regression and the top 10 metabolites ranked by random forest importance revealed distinct predictors for cardiovascular and all-cause mortality. For cardiovascular mortality, age (HR = 1.08, 95% CI: 1.02–1.14), Hb (HR = 0.95, 95% CI: 0.91–0.99), BUN (HR = 1.05, 95% CI: 1.02–1.08), HFD (HR = 0.11, 95% CI: 0.02–0.65), and 3’-adenosine monophosphate (3’-AMP, HR = 0.99, 95% CI: 0.99–0.99) emerged as key predictors. For all-cause mortality, age (HR = 1.09, 95% CI: 1.04–1.13), Hb (HR = 0.96, 95% CI: 0.93–0.99), PLT (HR = 0.99, 95% CI: 0.99–0.99), PTH (HR = 1.01, 95% CI: 1.01–1.01), CVD (HR = 2.89, 95% CI: 1.04–8.02), and uridine diphosphate (UDP, HR = 1.01, 95% CI: 1.01–1.01) were identified as critical risk factors (Tables 2 and 3).

**Table 2:** CVD mortality features selected by multivariate Cox regression analysis

Variables	CVD death Univariate analysis		CVD death Multivariate analysis	
	*P* value	HR (95%CI)	*P* value	HR (95%CI)
Age	< 0.001^*^	1.07 (1.04 ~ 1.10)	0.007^*^	1.08 (1.02 ~ 1.14)
Dialysis vintage	0.010^*^	1.01 (1.01 ~ 1.02)	0.872	1.00 (0.99 ~ 1.02)
Hb	0.016^*^	0.97 (0.95 ~ 0.99)	0.012^*^	0.95 (0.91 ~ 0.99)
BUN	0.038^*^	1.02 (1.01 ~ 1.04)	< 0.001^*^	1.05 (1.02 ~ 1.08)
PTH	0.020^*^	1.01 (1.01 ~ 1.01)	0.141	1.00 (1.00 ~ 1.00)
CVD	< 0.001^*^	4.07 (1.86 ~ 8.91)	0.129	2.62 (0.76 ~ 9.09)
Statins use	0.010^*^	3.64 (1.37 ~ 9.69)	0.801	1.27 (0.20 ~ 7.96)
HFD	0.034^*^	0.27 (0.08 ~ 0.91)	0.015^*^	0.11 (0.02 ~ 0.65)
cIMP	< 0.001^*^	1.01 (1.01 ~ 1.01)	0.999	1.00 (1.00 ~ 1.00)
Methionine sulfoxide	0.026^*^	1.01 (1.01 ~ 1.01)	0.411	1.00 (1.00 ~ 1.00)
3’-AMP	0.048^*^	0.99 (0.99 ~ 0.99)	0.018^*^	0.99 (0.99 ~ 0.99)
Glycerol 3-phosphate	0.462	1.00 (1.00 ~ 1.00)	-	-
UDP	0.003^*^	1.01 (1.01 ~ 1.01)	0.243	1.00 (1.00 ~ 1.00)
Phnylpyruvic acid	< 0.001^*^	1.01 (1.01 ~ 1.01)	0.459	1.00 (1.00 ~ 1.00)
Lauric acid	0.411	1.00 (1.00 ~ 1.00)	-	-
Adenosine 5’-phosphosulfate	0.477	1.00 (1.00 ~ 1.00)	-	-
Cysteinesulfinic acid	0.597	1.00 (1.00 ~ 1.00)	-	-
2-Hydroxybutyric acid	0.231	1.00 (1.00 ~ 1.00)	-	-

3’-AMP: 3’-adenosine monophosphate; BUN: blood urine nitrogen; CI: confidence interval; cIMP: cyclic inosine monophosphate; CVD: cardiovascular disease; Hb: hemoglobin; HFD: high-flux hemodialysis; HR: hazard ratio; PHT: parathyroid hormone; UDP: uridine diphosphate

**Table 3:** All-cause mortality features selected by multivariate Cox regression analysis

Variables	All-cause death Univariate analysis		All-cause death Multivariate analysis	
	*P* value	HR (95%CI)	*P* value	HR (95%CI)
Age	< 0.001^*^	1.07 (1.04 ~ 1.10)	< 0.001^*^	1.09 (1.04 ~ 1.13)
Hb	0.010^*^	0.97 (0.95 ~ 0.99)	0.008^*^	0.96 (0.93 ~ 0.99)
PLT	0.020^*^	0.99 (0.99 ~ 0.99)	0.012^*^	0.99 (0.99 ~ 0.99)
PTH	0.010^*^	1.01 (1.01 ~ 1.01)	0.033^*^	1.01 (1.01 ~ 1.01)
SBP	0.039^*^	1.02 (1.01 ~ 1.03)	0.099	1.02 (1.00 ~ 1.04)
DN	0.008^*^	3.66 (1.40 ~ 9.61)	0.679	1.69 (0.14 ~ 19.91)
DM	0.003^*^	3.34 (1.49 ~ 7.46)	0.331	2.75 (0.36 ~ 21.16)
CVD	< 0.001^*^	5.40 (2.72 ~ 10.74)	0.041^*^	2.89 (1.04 ~ 8.02)
Statins use	0.036^*^	2.79 (1.07 ~ 7.24)	0.640	1.40 (0.35 ~ 5.64)
HFD	0.041^*^	0.37 (0.14 ~ 0.96)	0.078	0.25 (0.05 ~ 1.17)
cIMP	< 0.001^*^	1.01 (1.01 ~ 1.01)	0.154	1.00 (1.00 ~ 1.00)
N-ε-acetyllysine-2	0.001^*^	1.01 (1.01 ~ 1.01)	0.369	1.00 (1.00 ~ 1.00)
UDP	0.002^*^	1.01 (1.01 ~ 1.01)	0.039^*^	1.01 (1.01 ~ 1.01)
dUMP	< 0.001^*^	1.01 (1.01 ~ 1.01)	0.100	1.00 (1.00 ~ 1.00)
2-Hydroxybutyric acid	0.051	1.00 (1.00 ~ 1.00)	-	-
Trans-Cinnamic acid	0.004^*^	1.01 (1.01 ~ 1.01)	0.078	1.00 (1.00 ~ 1.00)
Glycerol 3-phosphate	0.993	1.00 (1.00 ~ 1.00)	-	-
Phnylpyruvic acid	0.001^*^	1.01 (1.01 ~ 1.01)	0.181	1.00 (1.00 ~ 1.00)
N-Acetyl-β-alanine	0.011^*^	1.01 (1.01 ~ 1.01)	0.288	1.00 (1.00 ~ 1.00)
Barbituric acid	< 0.001^*^	1.01 (1.01 ~ 1.01)	0.787	1.00 (1.00 ~ 1.00)

CI: confidence interval; cIMP: cyclic inosine monophosphate; CVD: cardiovascular disease; DM: diabetes mellitus; DN: diabetic nephropathy; dUMP: deoxyuridine monophosphate; Hb: hemoglobin; HFD: high-flux hemodialysis; HR: hazard ratio; PLT: platelet; PHT: parathyroid hormone; SBP: systolic blood pressure; UDP: uridine diphosphate

### Risk models for CVD and all-cause mortality

**Fig. 2:**
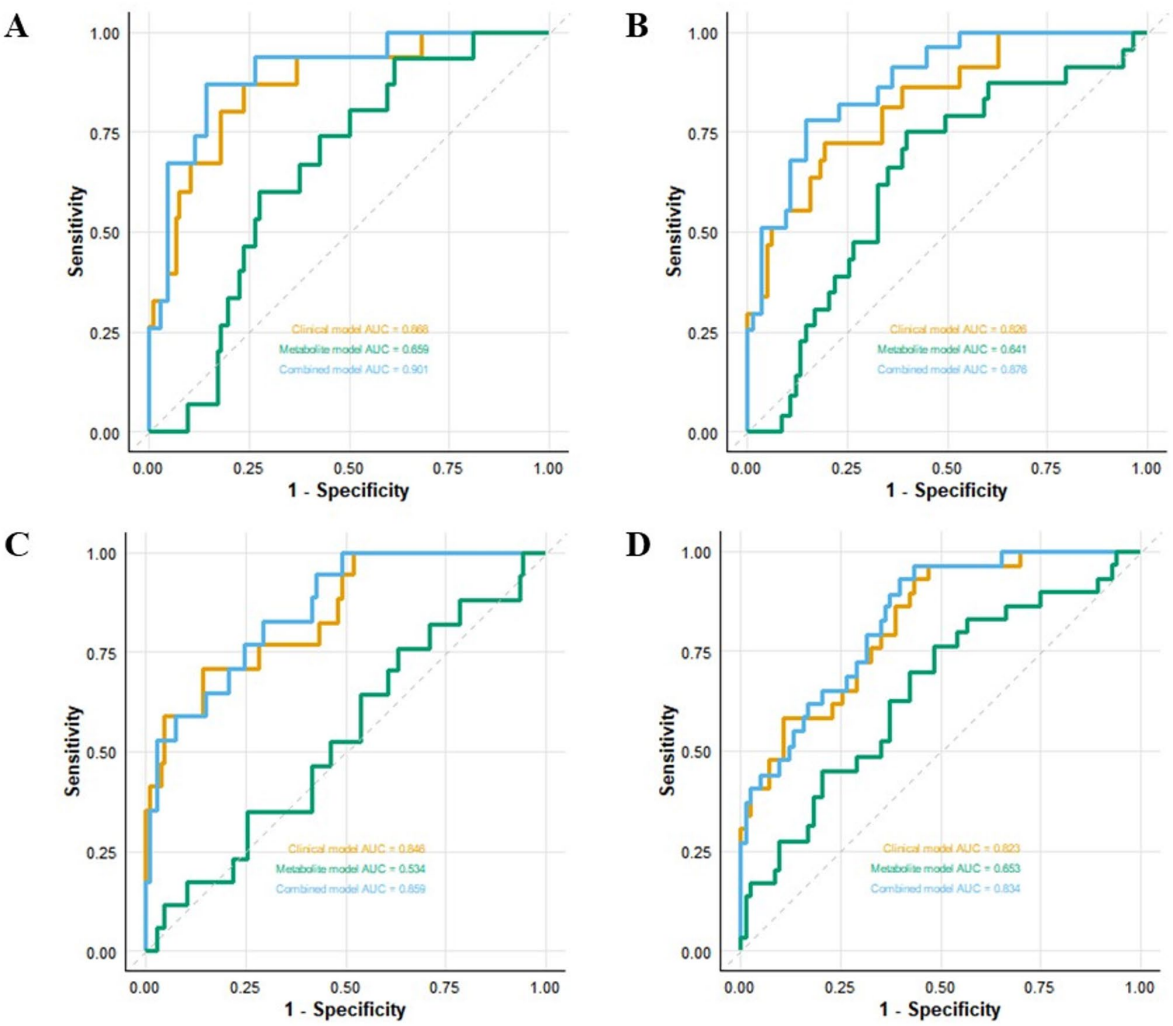
ROC curve comparisons of clinical, metabolomic, and combined models for predicting CVD and all-cause mortality in MHD patients: (**A**) 3-year CVD mortality; (**B**) 5-year CVD mortality; (**C**) 3-year all-cause mortality; (**D**) 5-year all-cause mortality

**Fig. 3:**
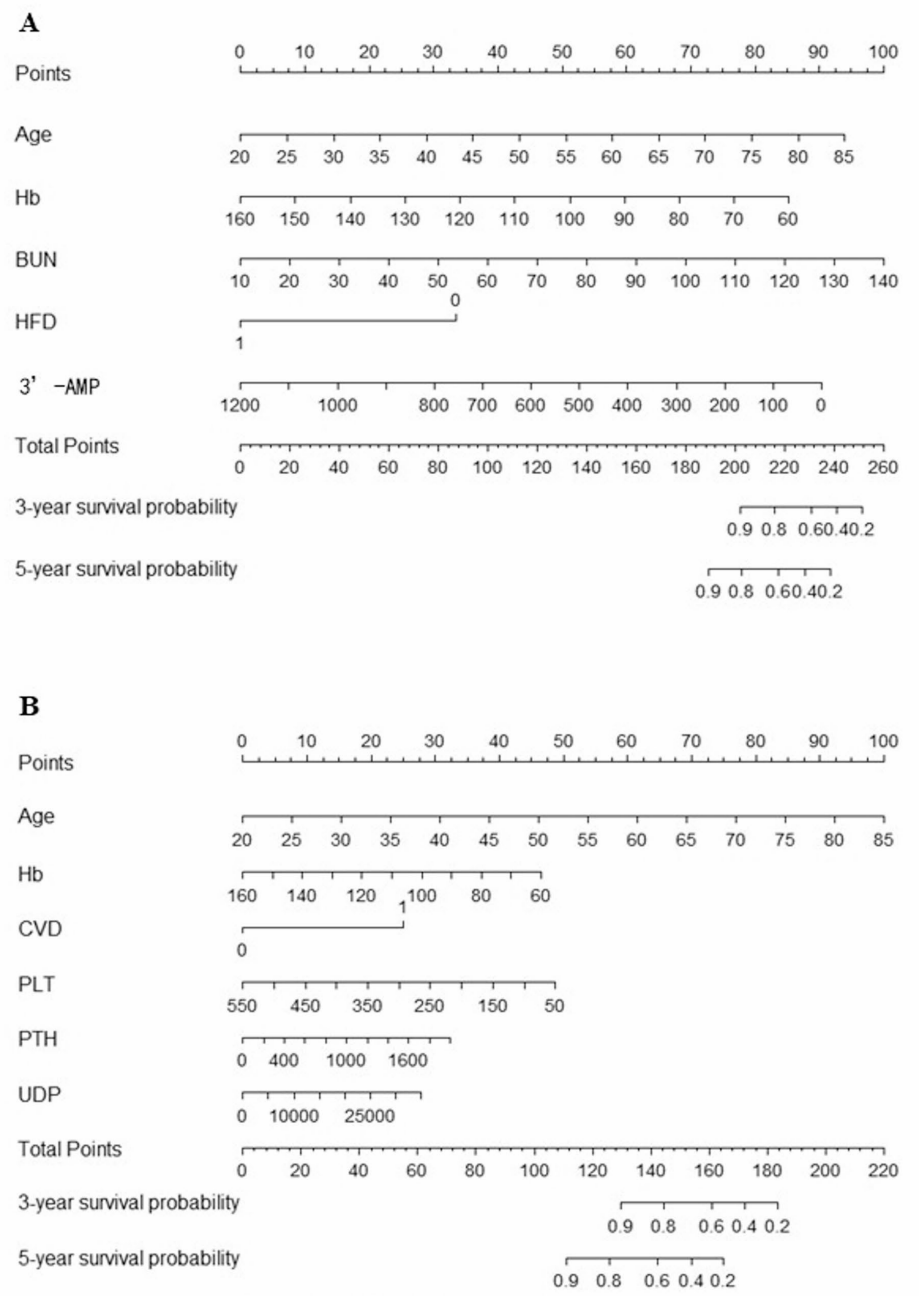
Nomogram for predicting CVD mortality and all-cause mortality risk in MHD patients: (**A**) Risk of CVD mortality; (**B**) Risk of all-cause mortality

**Fig. 4:**
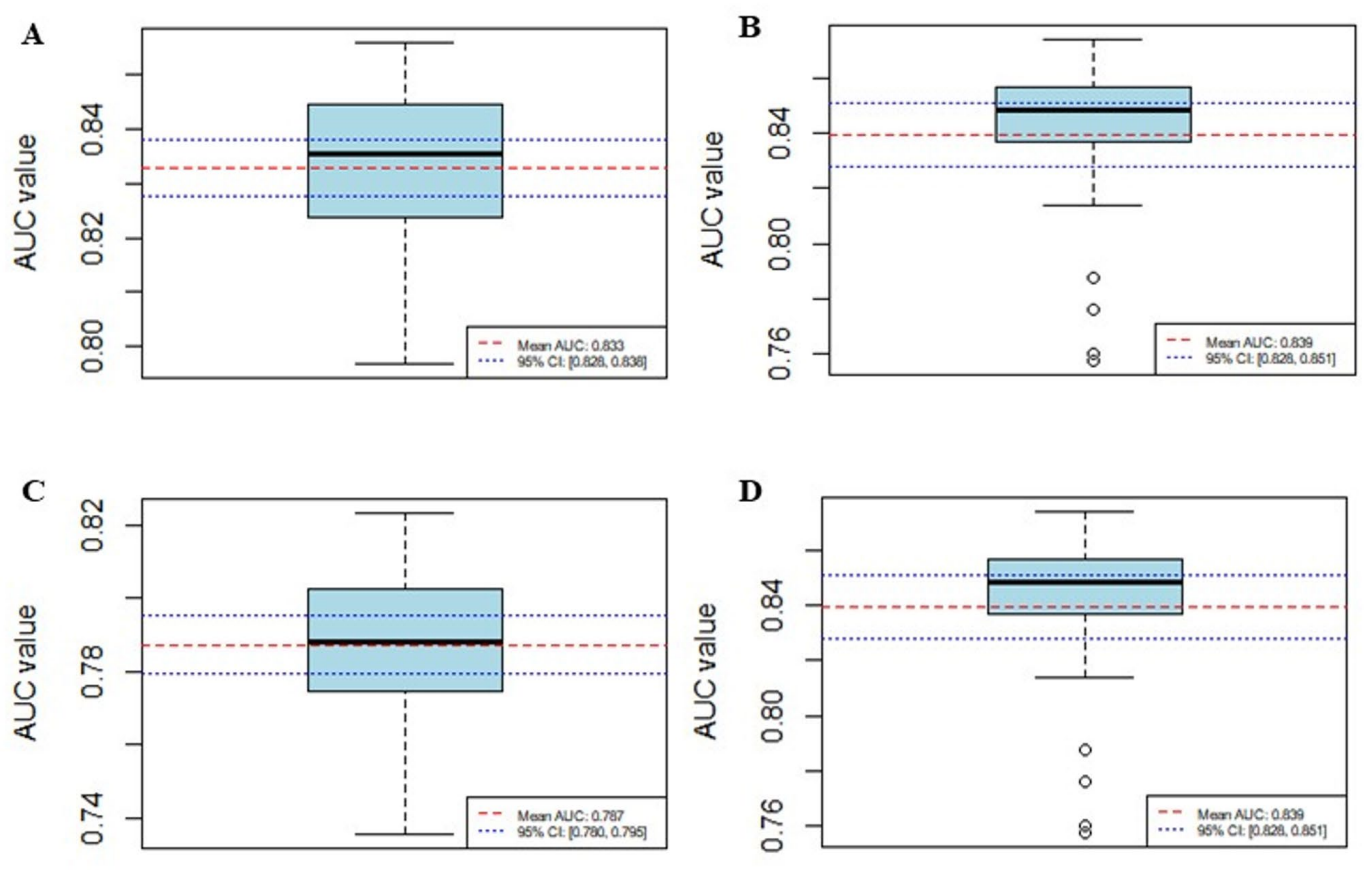
Mean AUC values of the combined model after cross-validation: (**A**) 3-year CVD mortality validation; (**B**) 5-year CVD mortality validation; (**C**) 3-year all-cause mortality validation; (**D**) 5-year all-cause mortality validation

During the model construction phase, this study developed three Cox regression risk prediction models based on distinct feature categories: a clinical model built using clinical features, a metabolite model established based on metabolite profiles, and a combined model integrating both clinical and metabolite features. The receiver operating characteristic (ROC) curves of each model are shown in Fig. 2. The results of the combined model are visualized as a nomogram, as shown in Fig. 3. After 30 rounds of 5-fold cross-validation, the average AUC results of the combined model are shown in Fig. 4.

For cardiovascular mortality prediction, the clinical model exhibited 3-year and 5-year AUC values of 0.868 and 0.826, respectively. The metabolite model showed 3-year and 5-year AUC values of 0.659 and 0.641. The combined model achieved 3-year and 5-year AUC values of 0.901 and 0.876. After cross-validation, the combined model yielded mean AUC values of 0.833 (95% CI: 0.828–0.838) for the 3-year prediction and 0.839 (95% CI: 0.828–0.851) for the 5-year prediction.

In all-cause mortality prediction, the clinical model demonstrated 3-year and 5-year AUC values of 0.846 and 0.823, respectively. The metabolite model displayed 3-year and 5-year AUC values of 0.534 and 0.653. The combined model attained 3-year and 5-year AUC values of 0.859 and 0.834. Following cross-validation, the combined model produced mean AUC values of 0.787 (95% CI: 0.780–0.795) for the 3-year prediction and 0.812 (95% CI: 0.808–0.816) for the 5-year prediction.

**Fig. 5:**
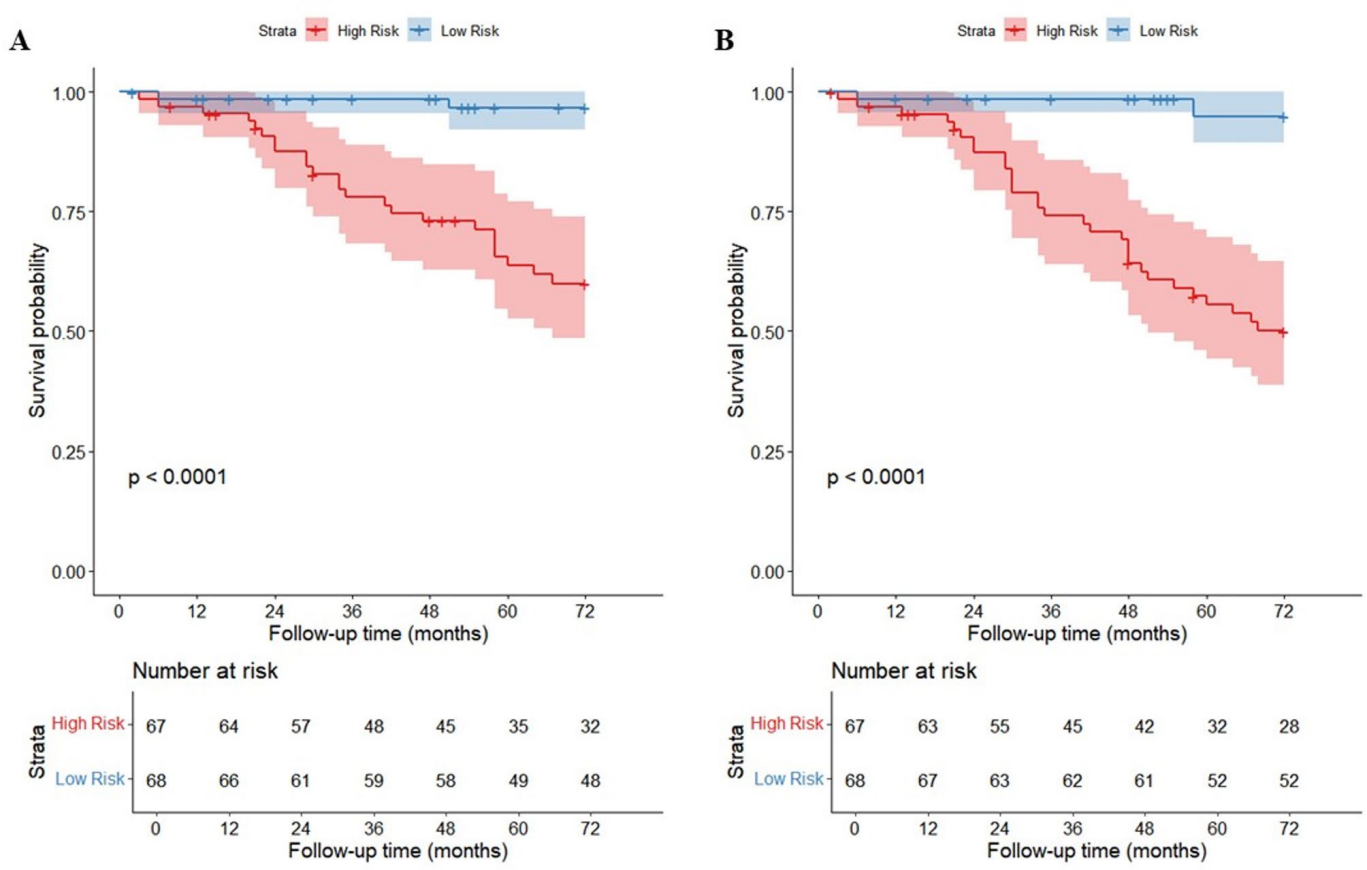
Cumulative risk curves based on combined model risk stratification: (**A**) High- and low-risk stratification for CVD mortality; (**B**) High- and low-risk stratification for all-cause mortality

Risk stratification based on the median risk score from the combined cardiovascular mortality model revealed a highly significant difference in cumulative mortality between high- and low-risk groups (*p* < 0.0001). Similarly, stratification for all-cause mortality showed significantly divergent survival curves between groups (*p* < 0.0001), as illustrated by the Kaplan-Meier (K-M) analysis in Fig. 5. The clinical baseline information of the high-risk and low-risk groups is shown in Supplementary Tables 1 and Supplementary Table 2.

## Discussion

This study developed risk prediction models for cardiovascular death and all-cause death in long-term hemodialysis patients. The combined model demonstrated superior predictive performance compared to single clinical or metabolic models, and cross-validation showed good stability of the combined model. The combined model for cardiovascular death prediction included five indicators: age, Hb, BUN, HFD, and 3’-AMP. The all-cause death prediction model comprised six indicators: age, Hb, PLT, PTH, CVD, and UDP. After stratification by median risk score, the cumulative mortality rate in the high-risk group was significantly higher than that in the low-risk group (*p* < 0.0001), confirming the clinical stratification value of the combined model.

In models of cardiovascular mortality and all-cause mortality, age serves as a common predictor, reflecting its role as a core risk factor in chronic disease progression and organ function decline, consistent with prior studies [14, 15]. Meanwhile, good control of anemia also brings multiple benefits, including lower mortality and hospitalization risks, reduced incidence of left ventricular hypertrophy, and improved cognitive function [16]. The inclusion of HFD suggests its potential benefit in clearing middle-molecular-weight toxins (such as β2-microglobulin) [17]. Moreover, in our previous study, we have confirmed that serum β2-microglobulin may be an important predictor of the risk of all-cause and CVD mortality in MHD patients [18]. Although our study suggests that an increase in urea levels is proportional to the risk of cardiovascular death, there is controversy [19]. In addition to the dialysis method and dialysis adequacy, the residual renal function of patients also needs to be further evaluated. MHD patients have a certain risk of thrombocytopenia, mainly due to immune or non-immune reactions triggered by the use of anticoagulants, as well as platelet activation, consumption, or destruction caused by poor biocompatibility of dialysis membranes [20]. A recent prospective cohort study has also confirmed that thrombocytopenia is associated with an increased risk of all-cause mortality [20]. Elevated PTH indicates the risk of calcium-phosphorus metabolism disorders and vascular calcification [21]. And a history of CVD further serves as a marker of systemic multi-system damage.

3’-AMP, as a metabolite of 2’,3’-cAMP, is further metabolized into adenosine through a series of reactions known as the 2’,3’-cAMP-adenosine pathway [22]. Adenosine exerts its effects by activating A_2A_ or A_2B_ receptors: activation of A₂A receptors inhibits the production of inflammatory cytokines TNF-α and CXCL10 to reduce inflammation, while activation of A₂B receptors suppresses vascular smooth muscle cell proliferation to slow the progression of atherosclerosis [23, 24]. Therefore, 3’-AMP may influence the risk of cardiovascular death in hemodialysis patients through anti-inflammatory and anti-atherosclerotic effects. In the all-cause mortality model, UDP is a critical positive inotropic factor involved in the development of cardiac diseases [25]. It can also promote the production of monocyte chemotactic factors via P2Y₆ receptors, exacerbating inflammatory responses [26]. Meanwhile, UDP may affect immune cell function by regulating intracellular calcium signaling and the release of inflammatory cytokines [27]. These mechanisms collectively may contribute to the increased risk of all-cause mortality in hemodialysis patients.

The clinical application of individual metabolites as biomarkers may be limited. The high baseline performance of the clinical model (AUC > 0.8) suggests that routine indicators (such as age and cardiovascular history) remain core predictive factors for prognosis, but the introduction of metabolites still contributes incremental value in the combined model. It is worth noting that the significant advantage of the combined model in predicting cardiovascular death indicates that the metabolite 3’-AMP complements pathological mechanisms not covered by traditional clinical indicators. However, the contribution of the metabolite UDP in predicting all-cause death is limited, possibly because all-cause death is driven by multiple factors (such as infection and gastrointestinal bleeding), and the specificity of existing metabolic biomarkers is insufficient. The slight decrease in AUC during cross-validation suggests vigilance against overfitting risks, but the overall model generalization ability remains acceptable. After grouping by the median risk score, the significant difference in cumulative incidence (*p* < 0.0001) indicates that the model can effectively distinguish high-risk and low-risk MHD patients, which is helpful for personalized monitoring and intervention.

This model has several remarkable advantages. Firstly, it reveals the distinct roles of metabolites in cardiovascular mortality and all-cause mortality among MHD patients, providing novel insights into their pathological mechanisms. Secondly, the model demonstrates extremely strong risk discrimination ability, offering a theoretical basis for early and precise interventions in high-risk patients. Finally, the risk visualization is achieved based on the nomogram, enabling rapid individual risk assessment without the need for complex calculations, which significantly improves the clinical practicality. However, this study also has several limitations. First, the predictive models were developed based on a single-center sample of MHD patients with a relatively small sample size, which may affect the models’ stability. Second, the clinical translation of metabolic markers (such as 3’-AMP and UDP) faces technical bottlenecks. Most medical institutions have not routinely performed tests for such substances, and targeted metabolomics technology has high detection costs. In the future, it will be necessary to develop rapid and low-cost detection methods to promote their clinical adoption. Third, potential predictive factors for CVD mortality in hemodialysis patients—such as N-terminal pro-B-type natriuretic peptide, inflammatory factors, β2-microglobulin, cardiac structure and function, and residual renal function—could not be included in the evaluation due to data missing. Finally, only internal cross-validation was performed without external validation.

## Conclusion

This study successfully constructed a death risk prediction model for hemodialysis patients by integrating clinical characteristics and metabolomics, confirming the significant advantage of the combined strategy in predicting cardiovascular death. The screening of metabolites 3’-AMP and UDP provides new clues for revealing the molecular mechanisms of hemodialysis-related metabolic disorders. In the future, it will be necessary to validate the model’s efficacy through multicenter cohorts and further explore the biological functions of key metabolites and their clinical intervention targets to promote precision risk management for MHD patients.

## Electronic supplementary material

Below is the link to the electronic supplementary material.

Supplementary Material 1

Supplementary Material 2

## Data Availability

The datasets used and/or analyzed during the current study are available from the corresponding author upon reasonable request.

## Abbreviations

3’-AMP: 3’-adenosine monophosphate
ALB: Albumin
ALP: Alkaline phosphatase
ALT: Alanine transaminase
AUC: Area under the curve
BUN: Blood urine nitrogen
Ca: Calcium
CE: Capillary electrophoresis
CI: Confidence interval
Cr: Creatinine
CVD: Cardiovascular disease
DM: Diabetes mellitus
ESRD: End-stage renal disease
Hb: Hemoglobin
HFD: High-flux hemodialysis
HR: Hazard ratio
IQR: Interquartile range
K: Potassium
K-M: Kaplan-Meier
MHD: Maintenance hemodialysis
Na: Sodium
P: Phosphorus
PLT: Platelet
PTH: Parathyroid hormone
ROC: Receiver operating characteristic
SBP: Systolic blood pressure
spKt/v: Single-pool Kt/V
TOF/MS: Time-of-flight mass spectrometry
UDP: Uridine diphosphate
