# Supplementary material for: Development of cardiovascular and all-cause mortality risk prediction models for maintenance hemodialysis patients based on metabolomics
Source: BMC Nephrol. 2025 Jul 10;26:372. doi: 10.1186/s12882-025-04291-0 (PMC12243235; doi:10.1186/s12882-025-04291-0)
Supplement: Supplementary file 1 — Supplementary Material 1 [file 12882_2025_4291_MOESM1_ESM.docx]

**Supplementary Table 1. Baseline clinical characteristics between high-risk and low-risk groups for cardiovascular death.**

| **Variables** | **High-Risk**  **(n=67)** | **Low-Risk**  **(n=68)** | **P High-Risk**  **vs Low-Risk** |
| --- | --- | --- | --- |
| Gender (Male) | 43 (64.18%) | 45 (66.18%) | 0.808 |
| Age (y) | 55.00 (50.00, 62.50) | 42.00 (36.00, 52.25) | <0.001^*^ |
| Dialysis vintage (m) | 85.00 (56.00, 123.50) | 69.50 (51.75, 83.50) | 0.014^*^ |
| BMI (kg/m^2^) | 21.85 (20.09, 25.03) | 23.43 (21.12, 26.59) | 0.045^*^ |
| Hb(g/L) | 110.00 (101.00, 115.00) | 116.00 (110.00, 121.00) | <0.001^*^ |
| PLT (*10^9^/L) | 175.00 (139.50, 206.50) | 201.50 (170.00, 232.00) | 0.002^*^ |
| ALT (U/L) | 13.00 (8.00, 17.00) | 11.00 (8.00, 16.00) | 0.391 |
| ALB (g/L) | 41.10 (39.50, 42.80) | 41.70 (40.45, 43.40) | 0.018^*^ |
| ALP (U/L) | 100.00 (76.00, 153.00) | 80.00 (66.00, 102.50) | 0.003^*^ |
| BUN (mmol/L) | 27.46 (23.79, 29.66) | 26.54 (23.95, 29.18) | 0.458 |
| Cr (μmol/L) | 972.00 (869.50, 1123.50) | 1133.50 (953.25,1309.50) | 0.002^*^ |
| K (mmol/L) | 5.06 (4.53, 5.87) | 4.96 (4.46, 5.52) | 0.175 |
| Na (mmol/L) | 134.74 ± 3.04 | 134.04 ± 3.38 | 0.208 |
| Ca (mmol/L) | 2.36 ± 0.18 | 2.37 ± 0.19 | 0.726 |
| P (mmol/L) | 2.03 (1.74, 2.44) | 2.12 (1.72, 2.46) | 0.857 |
| spKt/v | 1.34 (1.21, 1.49) | 1.34 (1.21, 1.53) | 0.790 |
| PTH (pg/mL) | 429.90 (227.70, 953.30) | 322.80 (147.00, 653.78) | 0.140 |
| SBP (mmHg) | 150.00 (138.00, 160.00) | 150.00 (140.00, 160.00) | 0.856 |
| DBP (mmHg) | 82.00 (80.00, 95.50) | 86.00 (80.00, 90.00) | 0.569 |
| DN | 4 (5.97%) | 5 (7.35%) | 1.000 |
| DM | 6 (8.96%) | 9 (13.24%) | 0.429 |
| Hypertension | 56 (83.58%) | 60 (88.24%) | 0.437 |
| CVD | 17 (25.37%) | 10 (14.71%) | 0.121 |
| Renal transplantation | 3 (4.48%) | 3 (4.41%) | 1.000 |
| Statins | 6 (8.96%) | 5 (7.35%) | 0.734 |
| Antihypertensive drugs | 46 (68.66%) | 49 (72.06%) | 0.665 |
| HFD | 7 (10.45%) | 35 (51.47%) | <0.001^*^ |
| IDH | 2 (2.99%) | 4 (5.88%) | 0.690 |
| 3-year mortality | 14(20.90%) | 1(1.47%) | <0.001^*^ |
| 5-year mortality | 22(32.84%) | 2(2.94%) | <0.001^*^ |

ALB: albumin; ALP: alkaline phosphatase; ALT: alanine aminotransferase; BMI: body mass index; BUN: blood urine nitrogen; CVD: cardiovascular disease; DBP: diastolic blood pressure; DM: diabetes mellitus; DN: diabetic nephropathy; Hb: Hemoglobin; HFD: high-flux hemodialysis; IDH: intradialysis hypotension; PLT: platelet; PHT: parathyroid hormone; SBP: systolic blood pressure; spKt/v: single-pool Kt/V.
