# Supplementary material for: Development of cardiovascular and all-cause mortality risk prediction models for maintenance hemodialysis patients based on metabolomics
Source: BMC Nephrol. 2025 Jul 10;26:372. doi: 10.1186/s12882-025-04291-0 (PMC12243235; doi:10.1186/s12882-025-04291-0)
Supplement: Supplementary file 2 — Supplementary Material 2 [file 12882_2025_4291_MOESM2_ESM.docx]

**Supplementary Table 2. Baseline clinical characteristics between high-risk and low-risk groups for all-cause death.**

| **Variables** | **High-Risk**  **(n=67)** | **Low-Risk**  **(n=68)** | **P High-Risk**  **vs Low-Risk** |
| --- | --- | --- | --- |
| Gender (Male) | 43 (64.18%) | 45 (66.18%) | 0.808 |
| Age (y) | 56.00 (51.00, 63.00) | 41.00 (35.75, 50.00) | <0.001^*^ |
| Dialysis vintage (m) | 87.00 (66.50, 126.00) | 61.00 (38.75, 82.00) | <0.001^*^ |
| BMI (kg/m^2^) | 21.85 (20.16, 25.16) | 23.20 (20.56, 26.57) | 0.159 |
| Hb(g/L) | 110.00 (99.00, 116.00) | 115.00 (110.00, 120.25) | <0.001^*^ |
| PLT (*10^9^/L) | 172.00 (130.00, 194.50) | 207.00 (182.50, 232.00) | <0.001^*^ |
| ALT (U/L) | 12.00 (8.00, 16.00) | 11.00 (7.00, 16.25) | 0.642 |
| ALB (g/L) | 40.52 ± 2.16 | 42.26 ± 1.94 | <0.001^*^ |
| ALP (U/L) | 98.00 (75.00, 167.50) | 85.00 (66.00, 102.25) | 0.002^*^ |
| BUN (mmol/L) | 27.46 (23.38, 29.45) | 26.48 (23.95, 29.90) | 0.802 |
| Cr (μmol/L) | 965.00 (828.00, 1064.00) | 1168.00 (1001.25, 1325.00) | <0.001^*^ |
| K (mmol/L) | 5.00 (4.51, 5.74) | 5.06 (4.46, 5.69) | 0.986 |
| Na (mmol/L) | 134.42 ± 3.16 | 134.36 ± 3.30 | 0.905 |
| Ca (mmol/L) | 2.37 ± 0.20 | 2.36 ± 0.17 | 0.770 |
| P (mmol/L) | 2.07 (1.73, 2.40) | 2.09 (1.75, 2.54) | 0.638 |
| spKt/v | 1.39 ± 0.24 | 1.34 ± 0.22 | 0.227 |
| PTH (pg/mL) | 450.10 (252.20, 1176.95) | 320.40 (126.28, 622.52) | 0.015^*^ |
| SBP (mmHg) | 151.00 (139.50, 160.00) | 150.00 (138.25, 160.00) | 0.281 |
| DBP (mmHg) | 84.00 (80.00, 92.50) | 84.00 (80.00, 90.00) | 0.731 |
| DN | 5 (7.46%) | 4 (5.88%) | 0.982 |
| DM | 9 (13.43%) | 6 (8.82%) | 0.394 |
| Hypertension | 59 (88.06%) | 57 (83.82%) | 0.479 |
| CVD | 24 (35.82) | 3 (4.41%) | <0.001^*^ |
| Renal transplantation | 3 (4.48%) | 3 (4.41%) | 1.000 |
| Statins | 7 (10.45%) | 4 (5.88%) | 0.332 |
| Antihypertensive drugs | 44 (65.67%) | 51 (75.00%) | 0.235 |
| HFD | 20 (29.85%) | 22 (32.35%) | 0.754 |
| IDH | 3 (4.48%) | 3 (4.41%) | 1.000 |
| 3-year mortality | 17 (25.37%) | 1 (1.47%) | <0.001^*^ |
| 5-year mortality | 27 (40.30%) | 3 (4.41%) | <0.001^*^ |

ALB: albumin; ALP: alkaline phosphatase; ALT: alanine aminotransferase; BMI: body mass index; BUN: blood urine nitrogen; CVD: cardiovascular disease; DBP: diastolic blood pressure; DM: diabetes mellitus; DN: diabetic nephropathy; Hb: Hemoglobin; HFD: high-flux hemodialysis; IDH: intradialysis hypotension; PLT: platelet; PHT: parathyroid hormone; SBP: systolic blood pressure; spKt/v: single-pool Kt/V.
